# Association between chromosome abnormities and prenatal diagnosis indicators screening in the second trimester of pregnancy

**DOI:** 10.1097/MD.0000000000034762

**Published:** 2023-09-01

**Authors:** Ci Pan, Zilong Li, Guomei Cheng, Xiaohua Luo, Fufang Nie, Jing Gao, Peifeng Yang

**Affiliations:** a Prenatal Diagnosis Center, The Third Affiliated Hospital of Zhengzhou University, Zhengzhou, China; b Jinan Pediatric Research Institute, Qilu Children’s Hospital, Cheeloo College of Medicine, Shandong University, Jinan, China; c Department of Obstetrics and Gynecology, The Third Affiliated Hospital of Zhengzhou University, Zhengzhou, China; d Department of Ultrasound, The Third Affiliated Hospital of Zhengzhou University, Zhengzhou, China.

**Keywords:** cell-free fetal DNA, chromosome abnormality, diagnostic indication, prenatal diagnosis, serum screening

## Abstract

This study aimed to explore the prenatal indicators in the second trimester of pregnancy and their association with chromosome abnormities (CA) to guide decisions toward invasive diagnostic procedures. Pregnant women who underwent prenatal screening and underwent amniocentesis in the second trimester in our Hospital between June 2017 and February 2019 were included in this retrospective cohort study. The reason for amniocentesis in prenatal screening and diagnoses was extracted from the charts. Finally, 3449 pregnant women were included. Of them, 181 were with CA confirmed by amniocentesis (i.e., the CA group), while 3268 were without CA (i.e., the non-CA group). Compared with the women in the non-CA group, those in the CA group were more likely to be older (30 [27,32] vs 29 [26,31], *P* < .001), had higher gestational weeks (20 [19,23] vs 19 [18,23], *P* = .008), an increased risk of advanced maternal age (AMA) (9.4% vs 2.2%, *P* < .001), had an increased risk of NIPT (IRN) (5.1% vs 1.9%, *P* < .001), had higher rates of a parental chromosome abnormality (PCA) (1.8% vs 0.9%, *P* = .002), and had increased risk of trisomy 21 (IRT21) (63.0% vs 45.3%, *P* < .001). AMA (OR = 4.22, 95% CI: 2.35–7.58, *P* < .001; AUC = 0.536), IRN (OR = 10.62, 95% CI: 6.66–16.94, *P* < .001; AUC = 0.589), PCA (OR = 4.77, 95% CI: 2.01–11.32, *P* < .001; AUC = 0.584), and IRT21 (OR = 0.67, 95% CI: 0.47–0.89, *P* = .008; AUC = 0.515) were independently associated with CA. AMA, IRN, IRT21, and PCA during the second trimester were independently associated with CA, but their predictive values for CA were relatively low. Combining those indicators may improve the predictive value.

## 1. Introduction

Fetal chromosome abnormality (CA) is one of the most common causes of birth defects and the key reason for prenatal screening (PS) and prenatal diagnosis (PD) in most countries. Presently, the primary reasons for PS are trisomy 21 (T21), trisomy 18 (T18), and neural tube defects (NTD). Serum screening in all pregnant women distinguishes high-risk groups who then undergo PD. In this way, the overuse of invasive PD (IPD) is effectively avoided, but there remains a chance of false positive and false negative results.^[1]^ In recent years, the use of IPD gradually decreased, but the yield of IPD increased, especially with the use of cell-free DNA.^[2]^

For decades, several studies tried to improve serum screening schemes. Various schemes were suggested, from the earliest double-screening, triple-screening, and quadruple-screening in the second trimester, to serum screening in the first trimester, combined with nuchal translucency (NT) screening.^[3]^ Noninvasive prenatal testing (NIPT) is considered the screening method with the highest accuracy. This method has been applied in clinics since 2011 and screens fetal chromosomes from cell-free fetal DNA in maternal peripheral blood.^[4]^ Nevertheless, primary screening does not usually involve NIPT because it is considered uneconomical, and serum screening remains preferred in most areas of the world.^[5]^ The high-risk factors requiring screening for Down’s syndrome due to T21 are well established,^[6]^ but it can often be more challenging to provide reasonable genetic counseling for fetal CA other than T21. It is important to understand how screening results and high-risk factors reflect the rate of chromosome abnormalities so that they can be discussed during genetic counseling.

One of our hospital's studies showed that serum screening combined with NT screening during the first and second trimesters was economical, with high detection and low false-positive rates.^[7]^ Nevertheless, in developing countries, including China, less than 20% of pregnant women have their first pregnancy screening before the 12th week, and most pregnant women undergo their first pregnancy screening in the 16th week, which misses the first-trimester window for serum and NT screening.^[8]^ Therefore, reasonable PS and PD are very important to low- and middle-income patients who have missed the first-trimester screening window and can only undergo serum screening in the second trimester. The initial diagnosis is usually confirmed by an IPD procedure.^[9]^ The main procedure for IPD is amniocentesis, but the procedure is not suitable for all pregnant women, can result in fetal loss, and causes anxiety to pregnant women.^[10]^ In pregnant women whose serum screening suggests a high risk of fetal CA, how this risk, combined with high-risk factors, indicates that they should undergo amniocentesis during the second trimester remains to be established. So, further understanding of the relationship between serum screening high-risk combined with risk factors and the occurrence of fetal CA is needed.

Therefore, this retrospective study explored the prenatal indicators in the second trimester of pregnancy and their association with CA to guide decisions toward invasive diagnostic procedures. This information could help genetic counselors provide women with vital information when deciding whether to undergo IPD.

## 2. Methods

### 2.1. Patients and study design

Pregnant women who underwent prenatal screening and underwent amniocentesis in the second trimester in Third Affiliated Hospital of Zhengzhou University between June 2017 and February 2019 were included in this retrospective cohort study. The second trimester was the period from week 14^0^ to week 27^+6^ of gestation.

The inclusion criteria were underwent amniocentesis in the second trimester (week 14^0^ to week 27^+6^ of gestation), underwent NIPT, and underwent amniocentesis. The exclusion criteria were multiple pregnancy or without any indication of amniocentesis but received amniocentesis because of anxiety.

The patients were classified as the CA and non-CA groups according to the amniocentesis results.

### 2.2. Data collection

The baseline data (maternal age and gestational age) of the pregnant women were collected from the hospital’s electronic medical record system. The reason for amniocentesis in prenatal screening was extracted from the charts, including advanced maternal age (AMA), increased risk of T21 (IRT21), increased risk of trisomy 18 (IRT18), abnormal ultrasonic finding (AUF),^[11]^ abnormal pregnancy history, parental CA (PCA), increased risk in NIPT (IRN), exposure history of teratogenic factors, and increased risk in NTD.

AMA was defined as expected delivery age ≥ 35 years old. IRT21 and IRT18 were determined automatically using the Lifecycle 3.2 software, according to α-fetoprotein, β-human chorionic gonadotrophin, and unconjugated estriol detected by a time-dependent immunofluorescence analysis system (Perkin-Elmer Life Sciences, Waltham, MA), combined with factors such as age, gestational age, previous reproductive history, with or without diabetes, and smoking or not.

The final diagnosis of CA was determined by amniocentesis, karyotype analysis, fluorescence in situ hybridization, and chromosomal microarray.

### 2.3. Statistical analysis

Statistical analysis was performed using SPSS 26.0 (IBM Corp., Armonk, NY) and R 4.1.2 (The R Project for Statistical Computing, www.r-project.org). The Kolmogorov-Smirnov test was used to assess the continuous variables for normal distribution. The data with a skewed distribution were presented as median (P25, P75) and compared using the Mann–Whitney *U* test. The categorical data were presented as n (%) and compared using the chi-square test. Characteristics showing a significant difference between groups were included in the multivariable logistic analysis (forward: LR). The diagnostic value of the final model and parameters included in the final model were determined by the receiver operating characteristics curve method and compared using Delong’s test. A 2-sided *P* < .05 was considered statistically different.

## 3. Results

In this study, the pregnant women with a singleton pregnancy who attended the Third Affiliated Hospital of Zhengzhou University were screened. Of these, 3449 pregnant women were finally included.

Of them, 181 were with CA confirmed by amniocentesis (i.e., the CA group), while 3268 were without CA (i.e., the non-CA group). Compared with the women in the non-CA group, those in the CA group were more likely to be older (30 [27,32] vs 29 [26,31], *P* < .001) and had higher gestational weeks (20 [19,23] vs 19 [18,23], *P* = .008), an increased risk of AMA (9.4% vs 2.2%, *P* < .001), an increased risk of NIPT (5.1% vs 1.9%, *P* < .001), and an increased risk of PCA (1.8% vs 0.9%, *P* = .002). On the other hand, the women in the non-CA group were more likely to have IRT21 (63.0% vs 45.3%, *P* < .001) (Table 1).

**Table 1:** The baseline characteristics and prenatal diagnoses of 2 groups.

Characteristics	CA (n = 181)	Non-CA (n = 3268)	*P*
Age (yr)	30 (27,32)	29 (26,31)	<.001
Gestational weeks	20 (19,23)	19 (18,23)	.008
AMA	17 (9.4)	73 (2.2)	<.001
IRT21	82 (45.3)	2060 (63.0)	<.001
IRT18	8 (4.4)	109 (3.3)	.433
IRNTD	2 (2.3)	42 (1.3)	>.999
AUF	79 (43.6)	1195 (36.6)	.055
APH	3 (3.4)	61 (1.9)	>.999
IRN	34 (5.1)	63 (1.9)	<.001
EHTF	0	8 (0.2)	>.999
PCA	7 (1.8)	28 (0.9)	.002

AMA = advanced maternal age, APH = abnormal pregnancy history, AUF = abnormal ultrasonic finding, CA = chromosome abnormality, EHTF = exposure history of teratogenic factors, IRN = increased risk in NIPT, IRNTD = increased risk in neural tube defects, IRT18 = increased risk of trisomy 18, IRT21 = increased risk of trisomy 21, PCA = parental chromosome abnormality.

The parameters with significant differences between the 2 groups were included in the multivariable logistics regression (forward: LR). The result showed that AMA (OR [95% CI] = 4.22 [2.35–7.58], *P* < .001), IRN (OR [95% CI] = 10.62 [6.66–16.94], *P* < .001), and PCA (OR [95% CI] = 4.77 [2.01–11.32], *P* < .001) were independently associated with a high risk of CA, while IRT21 (OR [95% CI] = 0.67 [0.47–0.89], *P* = .008) were independently associated with a lower risk of CA (Table 2).

**Table 2:** Multivariable analysis of prenatal diagnosis for CA.

Characteristics	OR	95% CI	*P*
AMA	4.220	2.348–7.584	<.001
IRT21	0.674	0.470–0.891	.008
IRN	10.617	6.656–16.935	<.001
PCA	4.770	2.009–11.322	<.001

AMA = advanced maternal age, CA = chromosome abnormality, CI = confidence interval, IRN = increased risk in NIPT, IRT21 = increased risk of trisomy 21, OR = odds ratio, PCA = parental chromosome abnormal.

**Figure 1.**
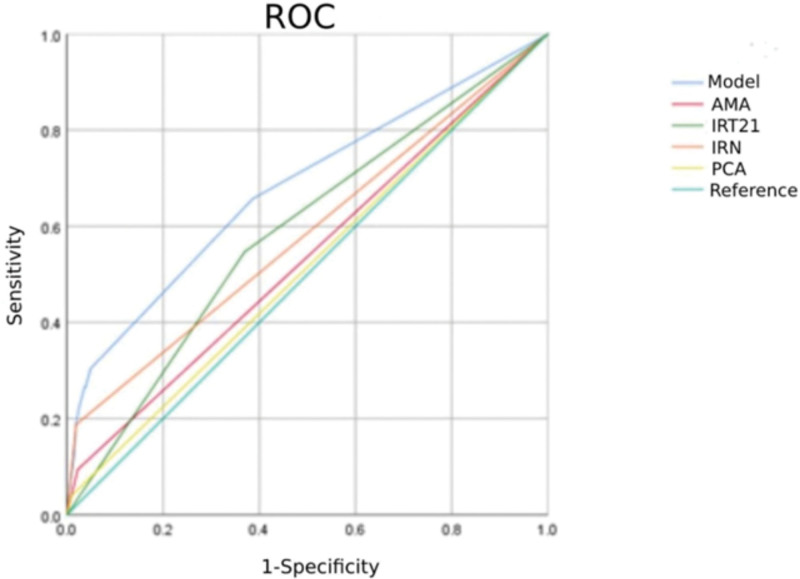
The ROC analysis of the final model and parameters included in the mode. ROC = receiver operating characteristics.

The receiver operating characteristics analysis was used to compare the diagnostic value of the parameters included in the final model. The areas under the curve (AUCs) of AMA, IRT21, IRN, and PCA were 0.536, 0.589, 0.584, and 0.515, respectively. While the model, including AMA, IRT21, IRN, and PCA, had a significantly higher AUC (AUC = 0.680) compared to those parameters alone (all *P* < .001) (Table 3; Fig. 1).

**Table 3:** ROC analyses of the factors associated with CA.

Model	Sensitivity	Specificity	AUC	*P* (Delong test)
Model	0.657	0.613	0.680	–
AMA	0.072	0.978	0.536	<.001
IRT21	0.177	0.630	0.589	<.001
IRN	0.169	0.981	0.584	<.001
PCA	0.030	0.991	0.515	<.001

AMA = advanced maternal age, AUC = area under the curve, CA = chromosome abnormality, IRN = increased risk in NIPT, IRT21 = increased risk of trisomy 21, PCA = parental chromosome abnormality, ROC = receiver operating characteristics.

## 4. Discussion

This study aimed to explore the diagnostic value of prenatal diagnosis indicators from pregnant women who underwent IPD after biochemical screening during the second trimester. The results suggested that AMA, IRN, and PCA were independently associated with a higher risk of CA in women who underwent IPD after biochemical screening during the second trimester, while IRT21 was independently associated with a lower risk. Therefore, this study provides some reference to clinical genetic counseling.

At present, amniocentesis is the primary method of IPD for chromosome abnormalities around the world. Generally, it is believed that the first 3 indications for undergoing amniocentesis are IRN, AMA, and AUF, resulting in a CAP from amniocentesis results of around 3.6%.^[12]^ In 2011, the karyotype analysis of 13,795 cases showed that most CA cases were aneuploidy except for those with the PCA indication.^[13]^ The present study showed that among IPD indications, IRT21 was the most common, followed by AUF, IRT18, AMA, abnormal pregnancy history, IRN, increased risk in NTD, and PCA. NIPT is the primary screening method for AMA women in China. A study of 12,365 cases from Henan of cultured amniotic fluid cells with karyotype analysis showed that the CAP was 3.46%.^[14]^ In this study, the CA rate was relatively high, which might be related to the increased use of serum screening and chromosomal microarray, which might have increased the detection of chromosome microdeletions and micro-duplications. Of course, local practice and reference criteria for PS and PD greatly influence the CAP. Of note, in the present study, the IRT21 was higher in the non-CA group than in the CA group. At the authors’ center, IRT21 and IRT18 are routinely determined automatically using the Lifecycle 3.2 software. This software was developed in the United States and there is a possibility that it might perform differently in Asian women, who have different genetic and biochemical characteristics than American women. There is no literature supporting a difference or not in the predictive value of the Lifecycle 3.2 algorithm in Chinese women, and it should be validated in the future. Nevertheless, contrary to American women, the results indicate that IRT21 may have a relatively lower predictive value for CA compared with other NIPT.

Currently, controversy exists regarding a prenatal screening-prenatal diagnosis for pregnant women of AMA. One side, the viewpoint that AMA is independently associated with CA hints at IPD being essential if missed diagnoses are to be avoided.^[15]^ The other side prenatal screening for pregnant women with AMA could effectively lessen unnecessary amniocentesis. In 2001, the American College of Obstetricians and Gynecologists recommended prenatal screening for pregnant women under 35 years old and IPD for pregnant women ≥35 years old. In 2008, a large multicenter study in China also reported that the amniocentesis ratio of pregnant women with AMA was reduced to 20.1% by serum screening in the second trimester.^[16]^ In 2013, it was suggested that the cutoff value of the increased risk of serum screening for pregnant women older than 35 years should be established in China.^[17]^ It was reported that most T21 and T18 could be detected using serum screening or serum screening combined with ultrasonic screening for pregnant women of AMA.^[18]^ The present study suggests that AMA was independently associated with fetal CA, which agrees with a Chinese study in 2008.^[16]^ Meanwhile, from the aspect of health economics, a mathematical model was applied to analyze the potency ratio of 3 serum screening schemes for pregnant women with AMA during the second trimester, implying that compared with direct amniocentesis, the scheme of amniocentesis for those with IRT21 showed the highest potency ratio and reduced costs by 74.8%.^[19]^ It provides important information for counseling pregnant women of AMA to guide their informed choice.

Since its clinical application, NIPT has altered the prenatal screening strategy and provided more options for prenatal screening. With a high ability to detect T21, T18, and T13 and its noninvasive character, NIPT is popular among pregnant women in high-risk groups. At the same time, NIPT is not popular among people in low-risk groups because of the high cost and chance of false-negative results. Currently, NIPT is more commonly used as secondary screening for pregnant women with high-risk factors unwilling to undertake IPD.^[20–22]^ In this study, IRN was independently associated with CA. Although the diseases screened by NIPT and serum screening are similar, the former is more sensitive and specific, and serum screening and NIPT are quite different from each other, even though both methods are screening rather than diagnostic tests.^[23]^

Of course, PCA indicates a higher risk of a CA being passed to the offspring or a higher risk of a failed meiosis event during gametogenesis.^[24,25]^ On the other hand, a study showed that PCA is associated with a high rate of miscarriage, but the chances of a healthy child were similar to non-PCA parents if a live birth is achieved.^[26]^ Additional studies are required to determine the impact of PCA.

Among the limitations of this study is the retrospective data collection. The number of patients with T18 serum screening in the data is relatively small, affecting the comparison of results. In addition, since not all IPD cases were collected during the same time period, trisomy 13 was not found in the data included in this study. It may be because trisomy 13 was detected in early pregnancy screening and NIPT screening, so it rarely appeared in the 2 IPD indicators of high risk in serum screening and abnormal ultrasound. Finally, the pregnancy outcomes could not be collected because several cases were terminated. Further studies, including prospective studies and postpartum follow-up, and larger cohort studies, are needed to determine the impact of the high risk of serum screening and ultrasound abnormalities during the second trimester on chromosomal abnormalities.

## 5. Conclusions

AMA, IRN, IRT21, and PCA during the second trimester are independently associated with CA, while their predictive values for CA were relatively low. Combining those indicators may improve the predictive value. Therefore, this study provided a reference to clinical genetic counseling.

## Author contributions

**Data curation:** Ci Pan, Zilong Li, Guomei Cheng, Xiaohua Luo, Fufang Nie, Jing Gao.

**Formal analysis:** Zilong Li, Guomei Cheng, Xiaohua Luo, Jing Gao.

**Project administration:** Ci Pan.

**Resources:** Fufang Nie.

**Writing – original draft:** Peifeng Yang.
